# Supplementary material for: Discovery and application of insertion-deletion (INDEL) polymorphisms for QTL mapping of early life-history traits in Atlantic salmon
Source: BMC Genomics. 2010 Mar 8;11:156. doi: 10.1186/1471-2164-11-156 (PMC2838853; doi:10.1186/1471-2164-11-156)
Supplement: Additional file 2 — Information on developed 76 locus single-run INDEL panel in Atlantic salmon. Information on fluorescence labeling, primer concentrations, PCR pooling and links to alignments, INDEL motifs and GENESCAN (Burge and Karlin 1997) predictions of genes/exons are available in html format. [file 1471-2164-11-156-S2.ZIP › Additionalfile2/snpsummary20742.html]

```
Cluster 11247 Contig 1

prev  Summary    Contig List  next
```

Size of Consensus sequence = 1382

Number of sequences = 4

Minimum redundancy = 1

Key

A gi|24394466|gb|CA064223.1|CA064223 ssalrga511331 mixed\_tissue Salmo salar cDNA, mRNA sequence  
B gi|117440460|gb|EG772683.1|EG772683 EST\_ssal\_evd\_48198 ssalevd thymus Salmo salar cDNA Salmo salar cDNA clone ssal\_evd\_564\_302\_rev 5', mRNA sequence  
C gi|117440459|gb|EG772682.1|EG772682 EST\_ssal\_evd\_48197 ssalevd thymus Salmo salar cDNA Salmo salar cDNA clone ssal\_evd\_564\_302\_fwd 3', mRNA sequence  
D gi|89866360|gb|DY722483.1|DY722483 EST\_ssal\_rgb2\_78222 ssalrgb2 mixed\_tissue Salmo salar cDNA Salmo salar cDNA clone ssal\_rgb2\_628\_215\_rev 5', mRNA sequence

7 SNPs detected

A B C D  cosegregation weighted

316 T - - .   7/7 75.00
317 A - - .   7/7 75.00
318 C - - .   7/7 75.00
319 A - - .   7/7 75.00
390 A G G .   7/7 75.00
828 . . G A   7/7 50.00
970 . . C A   7/7 50.00
